# Supplementary material for: Long ascending propriospinal neurons provide flexible, context-specific control of interlimb coordination
Source: eLife. 2020 Sep 9;9:e53565. doi: 10.7554/eLife.53565 (PMC7527236; doi:10.7554/eLife.53565)
Supplement: Supplementary file 1. — Time point comparisons reveal that silencing long ascending propriospinal neurons (LAPNs) did not affect the underlying foundation relationship between speed and stance duration, stride duration, and stride length at the fore- and hindlimbs, respectively. Running the same comparisons on the curated dataset of Dox-induced affected stepping yielded similar results (‘Dox-induced affected step’). Pearson correlation coefficients (r) and p values shown post-Bonferroni correction for multiple comparisons. [file elife-53565-supp1.docx]

**Supplementary File 1.**

|  | **Stance duration** | | | | **Stride duration** | | | | **Stride length** | | | |
| --- | --- | --- | --- | --- | --- | --- | --- | --- | --- | --- | --- | --- |
|  | **Forelimbs** | | **Hindlimbs** | | **Forelimbs** | | **Hindlimbs** | | **Forelimbs** | | **Hindlimbs** | |
| **Time point** | **r** | **p** | **r** | **p** | **r** | **p** | **r** | **p** | **r** | **p** | **r** | **p** |
| Baseline | -0.870 | p<0.001 | -0.758 | 0.027 | -0.746 | 0.034 | -0.715 | 0.060 | 0.862 | 0.002 | 0.792 | 0.012 |
| Pre-Dox1 | -0.902 | p<0.001 | -0.950 | p<0.001 | -0.890 | p<0.001 | -0.895 | p<0.001 | 0.872 | p=0.001 | 0.889 | p<0.001 |
| Dox1^On^-D3 | -0.892 | p<0.001 | -0.877 | p=0.001 | -0.874 | p=0.001 | -0.849 | 0.002 | 0.735 | 0.042 | 0.808 | 0.008 |
| Dox1^On^-D5 | -0.981 | p<0.001 | -0.957 | p<0.001 | -0.944 | p<0.001 | -0.911 | p<0.001 | 0.822 | 0.006 | 0.817 | 0.007 |
| Dox1^On^-D8 | -0.981 | p<0.001 | -0.971 | p<0.001 | -0.953 | p<0.001 | -0.952 | p<0.001 | 0.771 | 0.020 | 0.824 | P=0.005 |
| Dox^Off^ | -0.894 | p<0.001 | -0.930 | p<0.001 | -0.843 | 0.003 | -0.879 | p=0.001 | 0.781 | 0.016 | 0.673 | 0.117 |
| Sugar control | -0.934 | 0.021 | -0.962 | p=0.005 | -0.975 | 0.002 | -0.977 | 0.002 | 0.957 | 0.007 | 0.961 | 0.006 |
| Pre-Dox2 | -0.897 | p<0.001 | -0.837 | 0.004 | -0.867 | p=0.001 | -0.925 | p<0.001 | 0.445 | 0.128 | 0.753 | 0.030 |
| Dox2^On^-D3 | -0.950 | p<0.001 | -0.803 | p=0.010 | -0.913 | p<0.001 | -0.934 | p<0.001 | 0.645 | 0.174 | 0.710 | 0.065 |
| Dox2^On^-D5 | -0.870 | p=0.001 | -0.863 | p=0.001 | -0.880 | p=0.001 | -0.952 | p<0.001 | 0.642 | 0.180 | 0.736 | 0.040 |
| Dox-induced affected steps | -0.996 | 0.00033 | -0.956 | 0.0108 | -0.977 | 0.00405 | -0.95 | 0.0131 | 0.944 | 0.01590 | 0.905 | 0.0348 |
