# Supplementary material for: Long ascending propriospinal neurons provide flexible, context-specific control of interlimb coordination
Source: eLife. 2020 Sep 9;9:e53565. doi: 10.7554/eLife.53565 (PMC7527236; doi:10.7554/eLife.53565)
Supplement: Supplementary file 2. — Interlimb coordination data were analyzed using Watson’s non-parametric two-sample U (Orlovskiĭ et al., 1999) test (Critical value of Watson’s U2 = 0.1869; Appendix D, TableD.44) (Zar, 1974) [file elife-53565-supp2.docx]

**Supplementary File 2.**

|  |  |  | **Left-right forelimbs** | | **Left-right hindlimbs** | |
| --- | --- | --- | --- | --- | --- | --- |
| **Time point comparisons** | | | **p value** | **U^2^** | **p value** | **U^2^** |
| Baseline | vs | PD1 | 0.1<p<0.2 | 0.1199 | p>0.5 | -0.0759 |
|  |  | Dox1^On^-D3 | **0.01<p<0.02** | 0.2654 | 0.1<p<0.2 | 0.1298 |
|  |  | Dox1^On^-D5 | **0.02<p<0.05** | 0.2073 | **0.02<p<0.05** | 0.2262 |
|  |  | Dox1^On^-D8 | **0.001<p<0.002** | 0.3615 | **0.02<p<0.05** | 0.1906 |
|  |  | Dox^Off^ | p>0.5 | 0.0387 | p>0.5 | -0.1514 |
|  |  | Sugar control | p>0.5 | 0.0145 | p>0.5 | 0.0259 |
|  |  |  |  |  |  |  |
| Pre-Dox2 | vs | Dox2^On^-D3 | 0.1<p<0.2 | 0.1400 | **p<0.001** | 0.4337 |
|  |  | Dox2^On^-D5 | 0.1<p<0.2 | 0.1462 | **0.01<p<0.02** | 0.2399 |
|  |  |  |  |  |  |  |
| All Control | vs | All Dox | **p<0.001** | 0.672 | **p<0.001** | 1.4458 |
|  |  |  |  |  |  |  |
