# Supplementary material for: Long ascending propriospinal neurons provide flexible, context-specific control of interlimb coordination
Source: eLife. 2020 Sep 9;9:e53565. doi: 10.7554/eLife.53565 (PMC7527236; doi:10.7554/eLife.53565)
Supplement: Supplementary file 3. — Interlimb coordination data were analyzed using Watson’s non-parametric two-sample U (Orlovskiĭ et al., 1999) test (Critical value of Watson’s U2 = 0.1869; Appendix D, TableD.44) (Zar, 1974) [file elife-53565-supp3.docx]

**Supplementary File 3.**

| **Behavioral context** | **Watson’s test** | **Left-right FL** | **Left-right HL** | **Contra HL-FL** |
| --- | --- | --- | --- | --- |
| Overground locomotion | U^2^ | 0.6720 | 1.4458 | -0.3594 |
|  | p-value | *****p<0.001** | *****p<0.001** | p>0.5 |
| Treadmill locomotion | U^2^ | 0.0688 | 0.1513 | 0.0984 |
|  | p-value | p>0.5 | 0.10<p<0.20 | 0.2<p<0.5 |
| Exploratory-like locomotion | U^2^ | 0.1095 | -0.0307 | 0.1629 |
|  | p-value | 0.2<p<0.5 | p>0.5 | 0.05<p<0.10 |
| Overground locomotion on Sylgard coated surface | U^2^ | 0.2791 | 0.6128 | 0.3393 |
|  | p-value | ****0.005<p<0.01** | *****p<0.001** | ****0.002<p<0.005** |
| Overground locomotion on uncoated acrylic surface | U^2^ | -0.0655 | 0.0914 | 0.0542 |
|  | p-value | p>0.5 | 0.2<p<0.5 | p>0.5 |
| Overground locomotion, Sylgard coated vs uncoated acrylic (comparing control time points) | U^2^ | -0.5710 | 0.0600 | 0.0767 |
|  | p-value | p>0.5 | p>0.5 | 0.2<p<0.5 |
| Overground locomotion, Sylgard coated vs uncoated acrylic (comparing Dox^On^ time points) | U^2^ | 0.4412 | 0.4918 | 0.4496 |
|  | p-value | *****p<0.001** | *****p<0.001** | *****p<0.001** |
| Swimming | U^2^ | - | -24.1670 | - |
|  | p-value | - | p>0.5 | - |
